# An “Eight Perspectives” framework to describe complex psychobehavioral phenomena

**DOI:** 10.3389/fpsyg.2026.1847803

**Published:** 2026-06-23

**Authors:** Adam Bode, Marta Kowal

**Affiliations:** 1 Melbourne School of Psychological Sciences, University of Melbourne, Parkville, VIC, Australia; 2 IDN Being Human Lab—Institute of Psychology, University of Wroclaw, Wroclaw, Poland

**Keywords:** Eight Perspectives, evolutionary psychology, physical appearance enhancement, psychobehavioral phenomena, Tinbergen's Four Questions

## Abstract

Tinbergen's Four Questions is a widely used organizing framework for describing animal behavior that has also been applied to complex human psychobehavioral phenomena. However, it can be extended in several useful ways, making it a more comprehensive framework for the human social and behavioral sciences. To this end, we propose an Eight *Perspectives* framework that extends Tinbergen's Four Questions by specifying the placement and roles of ecology, emotions, cognitions, and behavior in a broader framework. The framework is designed to be useful, simple, and flexible. We demonstrate its application using the example of physical appearance enhancement, and show that it organizes findings in an easily interpretable manner, identifies gaps in knowledge, and invites collaboration across biology, ecology, psychology, and behavioral science.

## 1 Introduction

Evolutionary psychology is increasingly describing and explaining complex human psychobehavioral phenomena. The field has expanded from examining simple traits and behaviors to investigating multidimensional phenomena that span biology, ecology, psychology, and behavioral science. As the topics of investigation shift, so too do the ambitions of evolutionary psychologists, who often aim to provide more comprehensive accounts of such phenomena. To achieve this goal, evolutionary psychologists use organizing frameworks to guide their investigations.

One such organizing framework for describing behavior is Tinbergen's Four Questions (Tinbergen, 1963). This framework was designed to describe animal behavior but has also been adapted to describe human behavior (e.g., Kowal et al., 2024). One of the strengths of the Four Questions framework lies in its focus on both proximate and ultimate causes of behavior (Zietsch et al., 2021). Although frequently used in animal contexts (Kelly and Rose, 2024), when the phenomenon being described is a complex human psychobehavioral phenomenon, Tinbergen's Four Questions can be usefully augmented.

This perspective article proposes a novel framework for describing complex psychobehavioral phenomena. An “Eight Perspectives” framework extends Tinbergen's Four Questions to more comprehensively describe such phenomena, integrate findings, and build bridges between distinct research fields and disciplines. By approaching complex human psychobehavioral phenomena using Tinbergen's Four Questions and added dedicated consideration of ecology, psychology, and behavioral science, evolutionary psychologists and others may be able to provide more comprehensive descriptions of many important aspects of human nature.

## 2 Complex human psychobehavioral phenomena

Over the past 40 years, evolutionary psychologists and others have sought to describe and explain complex human psychobehavioral phenomena. These phenomena are “complex” in that what is being investigated has multiple interacting components (e.g., biology, psychology, behavior) and cannot be reduced to a single trait or behavior. These phenomena also involve particularly human aspects (e.g., language, self-report, and cultural transmission). Such aspects are not present or easily accessible in animal species. These phenomena are also “psychobehavioral”, in that the phenomena being investigated integrate psychological (i.e., emotions and cognitions) and behavioral components. Examples of these phenomena include attachment (Simpson and Belsky, 2016), romantic love (Bode and Kushnick, 2021), paternal care (Geary, 2000), and grief (Archer, 1999).

Describing and explaining complex human psychobehavioral phenomena requires consideration of both ultimate and proximate perspectives, as well as their integration. Tinbergen's Four Questions is one framework that does this, but is underused in the psychological and behavioral sciences. Some examples of Tinbergen's Four Questions being fruitfully applied to complex human psychobehavioral phenomena include romantic love (Bode and Kushnick, 2021), sleep (Bode and Kuula, 2021), female sexual orientation (Luoto et al., 2019) and religious ritual (Legare and Nielsen, 2020). One reason that this framework is not employed as often as it could be—despite its apparent utility—is that it is not regularly usefully broadened to enhance its applicability to complex psychobehavioral phenomena.

## 3 Tinbergen's four questions

Tinbergen's Four Questions (Tinbergen, 1963), as they came to be known, is an organizing framework for describing the biology of animal behavior. Tinbergen (1963) essentially argued that a comprehensive understanding of any behavior requires answering four complementary questions about its causation, survival value, ontogeny, and evolution. This required integration of four perspectives across two levels of explanation (ultimate and proximate; see Mayr, 1961). The framework has been widely recognized as seminal and extended in the behavioral and evolutionary sciences literature (Bateson and Laland, 2013; Bergman and Beehner, 2022). It possesses a number of features that could potentially be useful when studying complex human psychobehavioral phenomena, but there are also several ways in which it can be usefully extended and augmented (see also Al-Shawaf, 2025).

### 3.1 The benefits of Tinbergen's Four Questions for considering complex human psychobehavioral phenomena

Tinbergen's Four Questions has two primary benefits when applied to psychobehavioral phenomena. First, it requires consideration of four complementary questions crossing both ultimate and proximate levels of explanation (see Zietsch et al., 2021). Second, it places the study of such phenomena within a biological vocabulary shared across disciplines, facilitating interdisciplinary collaboration (e.g., Legare and Nielsen, 2020).

### 3.2 Ways that Tinbergen's Four Questions can be usefully broadened for considering complex human psychobehavioral phenomena

Despite its many benefits, there are several ways that Tinbergen's Four Questions can be profitably broadened to help describe complex human psychobehavioral phenomena. First, Tinbergen's Four Questions were initially designed for animal behavior, and therefore the framework makes no reference to psychology, whether it be emotions or cognitions. Some researchers using Tinbergen's Four Questions for complex human psychobehavioral phenomena have extended the framework and classified psychology as falling within mechanisms (e.g., Bode and Kushnick, 2021; Bode and Kuula, 2021; Legare and Nielsen, 2020; Luoto et al., 2019). This is a reasonable place for it, but this creates a bit of a catch-all approach with conceptually and practically distinct features falling within the one section. While this could in principle be addressed by using subsections within mechanisms, a more comprehensive approach would be to consider emotions and cognitions in any framework as *both* mechanisms and phenotypic outcomes.

In complex human psychobehavioral phenomena, the cognitions and emotions are often so fundamental to the phenomenon that they justify classification as a phenotypic outcome just as much as they do a cause of behavior. The concept of phenotype has been extended over time from simply physical characteristics to behavior (e.g., Goodey, 2006), to psychological features (e.g., cognitive phenotypes, Brody, 1994; psychopathology, Tsuang et al., 1993). At its simplest, a phenotype is a measurable and detectable feature resulting from an interaction between genes and the environment (Gottesman and Gould, 2003). We acknowledge that cognitions and emotions can also be conceptualized as mechanisms at the information-processing level. However, for the purpose of describing psychobehavioral phenomena, we propose that they can often be usefully treated as phenotypic outcomes worthy of explanation in their own right—not just causes of behavior.

A second way that Tinbergen's Four Questions can be usefully broadened when applied to complex human psychobehavioral phenomena is that the framework treats behavior as explanandum of the entire framework. In ethology, behavior is defined as observable movement and serves as the phenomenon to be explained (Tinbergen, 1963). In complex human psychobehavioral phenomena, behavior is part of the phenotypic expression along with emotions and cognitions and warrants consideration to the same degree as those other perspectives. Previous efforts applying Tinbergen's Four Questions (Bode and Kushnick, 2021) have detailed aspects of behavior across all four perspectives, rather than being coherently considered in its own perspective. This limits the role that behavioral science plays in describing and explaining complex human psychobehavioral phenomena.

A third key consideration is that the Four Questions framework says little about ecology because, for animal behavior, the natural environment is the context in which survival value is assessed. In complex human psychobehavioral phenomena, however, ecology is particularly influential in shaping phenotypic outcomes (Nettle, 2009), and constitutes its own analytical tradition. For example, ecology provides insight into the impact of cross-cultural variation (Amir and Pitt, 2026; Sorokowski et al., 2023) or socioecological predictors (Buss et al., 1990) on such phenomena. These are questions that ecologists, human behavioral ecologists, and cross-cultural psychologists pursue with their own methods and theoretical angles. There is a need to consider aspects of ecology across the four biological perspectives. For example, it is appropriate to consider ecological factors operating as selective pressures in the ancestral past under evolutionary functions. However, this does not prevent a specific focus on ecological influences on the modern expression of a phenomenon. Ecology can therefore be viewed as an important set of inputs and influences operating across multiple timescales, cutting across multiple levels of analysis in the framework. This is implicit in the Four Questions framework, but it is worth making explicit. Doing so ensures that researchers from ecological traditions (i.e., human behavioral ecology and cross-cultural psychology) can see their contributions clearly represented within the framework.

## 4 Eight perspectives for considering complex human psychobehavioral phenomena

### 4.1 . The aims

Amending and extending Tinbergen's Four Questions is not unprecedented behavior (Al-Shawaf, 2025; Bateson and Laland, 2013; Bergman and Beehner, 2022; Konner, 2021; Nesse, 2013). To capitalize on the utility of Tinbergen's Four Questions and extend it when applied to complex human psychobehavioral phenomena, we propose an Eight *Perspectives* framework which extends and operationalizes that framework in a manner that is useful, simple, and flexible, and which invites collaboration, multidisciplinarity, and interdisciplinarity. By *useful*, we mean that the framework organizes findings in a way that evolutionary psychologists actually employ, and that the resulting organization can inform the generation of new predictions and explanations by researchers. We want it to be practical, not just theoretically elegant. By *simple*, we mean that the framework is parsimonious—Eight Perspectives is enough to cover the terrain but not so burdensome as to hinder its application or invite unnecessary conceptual bloat. By *flexible*, we mean that the framework can be applied to myriad complex human psychobehavioral phenomena. It does not need to be applied rigidly. We do not claim that this is the only useful framework for understanding psychobehavioral phenomena, and we expect it to complement rather than replace existing approaches. Such an approach differs from other efforts to extend or augment Tinbergen's Four Questions which have largely attempted to consider the biology of behavior (Bateson and Laland, 2013; Bergman and Beehner, 2022; Konner, 2021; Nesse, 2013), with one notable exception focused on integrating levels of analysis for psychology (Al-Shawaf, 2025). We are focused explicitly on complex human psychobehavioral phenomena. We also wish to emphasize very clearly that the Eight Perspectives framework is not intended as a *replacement* of Tinbergen's Four Questions, but rather a useful extension that is specifically designed to apply to complex human emotions, cognitions, and behaviors.

### 4.2 . Components

Before describing each perspective, we clarify what kind of framework Eight Perspectives is. The explanandum is the complex human psychobehavioral phenomenon itself, not behavior alone; behavior is part of the phenomenon's phenotypic expression, alongside emotions and cognitions. The framework therefore combines explanatory perspectives drawn from Tinbergen (i.e., evolutionary history, evolutionary function, development, [biological] mechanisms) with perspectives that characterize the phenomenon's phenotypic expression (emotions, cognitions, behavior) and the ecological processes that shape it. In this sense, Eight Perspectives should be understood as an organizational extension of Tinbergen's Four Questions rather than a competing causal taxonomy. Tinbergen's four explanatory categories remain logically sufficient for causal explanation, while the additional perspectives may provide an alternative and in some cases more useful framework for describing complex human psychobehavioral phenomena.

**Figure 1:**
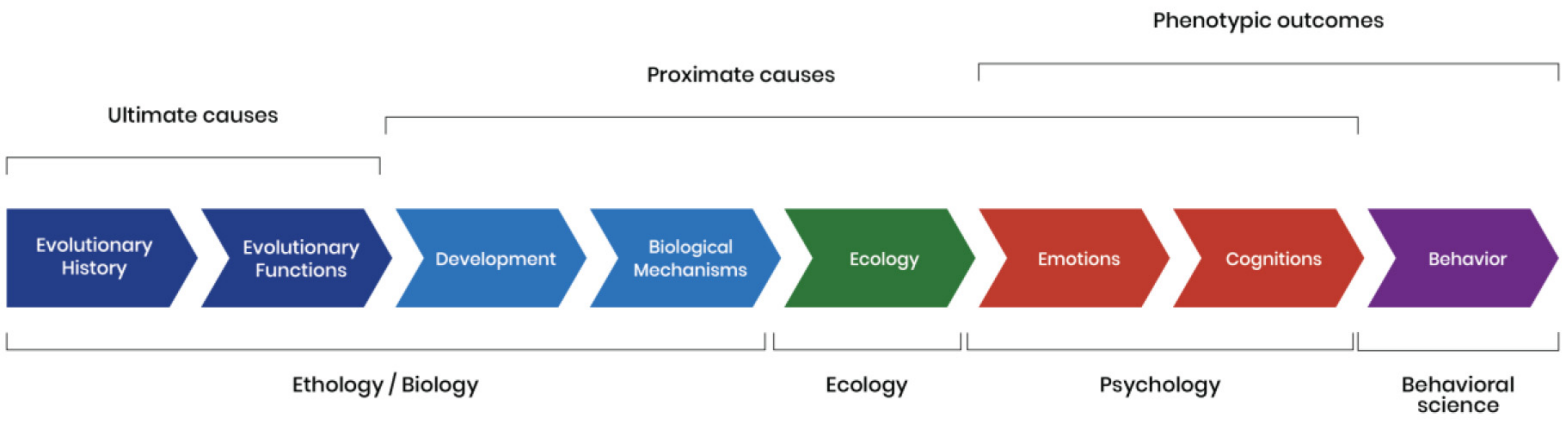
“Eight Perspectives” to describe complex psychobehavioral phenomena.

The Eight Perspectives framework (Figure 1) retains Tinbergen's Four Questions as a foundational base, to which it adds ecology, emotions, cognitions, and behavior. These are perspectives, and not levels of explanation as outlined by Mayr (1961), although the framework does recognize both ultimate and proximate causes of phenotypic outcomes. We use the term “perspectives” to denote distinct vantage points from which a phenomenon can be examined, rather than implying that each perspective has the same ontological or explanatory status. Figure 1 presents Eight Perspectives. These include evolutionary history and evolutionary functions (ultimate causes), development and mechanisms (proximate causes), ecology (a source of inputs that cuts across timescales and levels of analysis), emotions and cognitions (which are treated as both proximate causes of behavior *and* phenotypic outcomes in their own right), and behavior (phenotypic outcomes). These perspectives are aligned with and informed by the scientific disciplines of biology/ethology, ecology, psychology, and behavioral science. We do note that not all perspectives will be equally relevant to all phenomena, and there may be circumstances when additional considerations are justified. The order in which these perspectives are presented can be adjusted to provide the most intuitive narrative for the phenomenon being described.

*Evolutionary history* considers the phylogenetic origins of the phenomenon, including its timing of emergence, universality, and the processes involved in its evolution. *Evolutionary function* considers the selective pressures that shaped the phenomenon—what problem the system evolved to solve. *Development* considers how the phenomenon emerges ontogenetically and changes across the lifespan. *Biological Mechanisms* considers the neurobiological, endocrinological, and genetic bases that underlie the phenomenon. We use the modifier “Biological” to make explicit that emotions and cognitions—also proximate causes of behavior in the Tinbergenian sense—are addressed in their own perspectives. This refines rather than replaces Tinbergen's causation question, distributing its content across three perspectives where each can be addressed in its own terms. It differs from previous applications of Tinbergen's Four Questions to psychobehavioral phenomena, which placed psychological mechanisms under causation (e.g., Bode and Kushnick, 2021; Legare and Nielsen, 2020), and aligns with Tinbergen (1963), who himself excluded subjective experience from causal explanation. *Ecology* considers the dynamic processes through which phenotypes emerge from interactions between organisms and their physical, social, and cultural environments (e.g., socioecological variation, cross-cultural variation) operating across evolutionary, ontogenetic, and immediate timescales. Aspects of ecology can reasonably be considered under other perspectives (e.g., frequency dependence, developmental responses), and researchers may choose to do so where it suits the phenomenon under study. However, ecology also constitutes a coherent analytical tradition (human behavioral ecology, cross-cultural psychology) that explains phenotypes as emergent products of organism–environment dynamics; treating it as a dedicated perspective preserves that explanatory work. *Emotions* considers the affective experiences and drivers of the phenomenon. Emotions can also be considered in the context of being mechanisms that facilitate cognition or behavior. *Cognitions* considers the characteristic patterns of thought associated with the phenomenon. Cognition can also be considered in the context of being mechanisms that facilitate emotion or behavior. *Behavior* considers the observable actions and practices that constitute the behavioral expression of the phenomenon.

## 5 Application of eight perspectives to physical appearance enhancement

As an illustrative example, we show how the Eight Perspectives framework provides a useful framework for analyzing the phenomenon of physical appearance enhancement (see Table 1). Consideration of the evolutionary history of physical appearance enhancement revealed findings about universality, the timing of its emergence in the *Homo* lineage, and evolutionary processes. Consideration of evolutionary functions identified the selective pressures that may have shaped the phenomenon, including intersexual and intrasexual competition. A developmental perspective revealed the ontogenetic trajectory of appearance-related preferences and behaviors from infancy through adulthood. Consideration of mechanisms identified neurobiological substrates, hormonal involvement, and genetic contributions. An ecological perspective revealed how cultural and environmental factors likely shape cross-cultural variation in appearance enhancement investment. Consideration of emotions identified specific affective experiences associated with the phenomenon, including their valence and intensity. A cognitive perspective revealed characteristic patterns of thought, including social comparison, internalization of beauty ideals, and rumination. Finally, consideration of behavior identified the specific forms, costs, and sex and cultural differences in appearance enhancement practices. See Table 1 for a summary, along with references for each.

**Table 1:** Perspectives on physical appearance enhancement.

Perspective	Key findings	Key references
Evolutionary history	•Probably a human universal •Evidence of ochre-based pigment use in Neanderthals 200–250 thousand years ago (Kya) and 164 Kya in early sapiens •Evidence of personal ornaments in humans 70–120 Kya •Intra- and inter-sexual competition probably played a role	• Kowal et al., 2022 • Roebroeks et al., 2012 • Marean et al., 2007 • d'Errico et al., 2009 • Davis and Arnocky, 2022
Evolutionary functions	•Intersexual selection (mate attraction and mate retention) •Intrasexual competition (outcompeting rivals) •Signaling mate quality (health, fertility, genetic quality) •Signaling status •Securing status and resources •Social affiliation and group membership	• Davis and Arnocky, 2022 • Kowal et al., 2022 • Dixson, 2022 • Blake, 2022
Development	•Preference for attractive faces probably innate, as evidenced by longer gaze duration on more vs. less attractive faces by infants even a few months old •Body surveillance and often dissatisfaction emerge in early childhood •Body dissatisfaction is relatively stable from mid-adolescence through adulthood •Intense appearance-enhancement behaviors emerge and elaborate following puberty	• Langlois et al., 1987 • Bucchianeri et al., 2013 • Wang et al., 2019 • Tatangelo et al., 2016
Biological mechanisms	•Different neural response to more vs. less attractive faces •Sex hormones (testosterone, estrogen) modulate attractiveness preferences •Genes play a role in body dissatisfaction and appearance-related eating pathology	• Chatterjee et al., 2009 • Winston et al., 2007 • Keski-Rahkonen et al., 2005 • Puts et al., 2013
Ecology	•Cultural (e.g., gender equality) and environmental (e.g., pathogen stress) factors are associated with enhancement investment •Western beauty standards and means to enhance attractiveness are globalizing •Modern social media environments expose individuals to supernormal stimuli (e.g., digitally altered images) that ancestral mechanisms were not designed for	• Kowal et al., 2022 • Kowal et al., 2024 • Swami, 2015
Emotions	•Myriad particular emotions (e.g., body shame, body dissatisfaction, anxiety, envy) •Intense emotions of both positive (proud) and negative (envy) valence	• Arnocky et al., 2016 • Frederick and Reynolds, 2022
Cognition	•Frequent social comparisons •Internalization of prevalent beauty ideals •Cognitive rumination of discrepancy between oneself and more physically attractive others	• Thompson and Stice, 2001 • Myers and Crowther, 2009 • Dondzilo et al., 2023 • McComb and Mills, 2021
Behavior	•Physical appearance enhancement is highly time and resources intensive •Characteristic types of physical appearance enhancement vary between men and women and across cultures •Typical forms include cosmetics, clothing, hairstyling, dieting, exercise, body modification, and hygiene •Behavior is context-sensitive (e.g., physical appearance enhancement intensifies during courtship)	• Kowal et al., 2022 • Kowal et al., 2024 • Davis and Arnocky, 2022

## 6 Discussion

### 6.1 The Eight perspectives framework is useful, simple, and flexible

Applying Eight Perspectives to physical appearance enhancement demonstrates that the framework is useful, simple, and flexible. It is useful because it has organized findings about physical appearance enhancement in a way that is easily interpretable and fits on a single page. A cursory glance at the findings highlights that relatively little is known about development and mechanisms—which means that the framework has also identified priority areas for future research. This example also demonstrates that the framework is simple. The result required no specialized theoretical apparatus, application, or interpretation. Finally, this example also highlights the flexibility of the framework. Physical appearance enhancement was chosen as the illustrative case here, but the framework's structure (i.e., biological, ecological, psychological, and behavioral perspectives) applies equally well to phenomena with different profiles, such as jealousy (where emotions dominate), grief (where development across the lifespan is central), or aggression (where ecology and behavior are most prominent).

### 6.2 General benefits of eight perspectives

There are a number of benefits to using Eight Perspectives to consider complex human psychobehavioral phenomena. The first is that it usefully extends Tinbergen's Four Questions framework in a way that permits even more comprehensive description, and in a way that is maximally applicable to complex human behaviors. Psychology (i.e., emotions and cognitions) and ecology are represented through their own perspectives, while behavior is addressed in detail. We suggest that this multifaceted framework invites collaboration, multidisciplinarity, and interdisciplinarity. The Eight Perspectives framework can also be usefully applied to a wide variety of topics, such as jealousy, aggression, eating behavior, or grief, to name just a few. The Eight Perspectives framework can also scale with the size of the project being undertaken. For simple projects or phenomena, it can fit on to a single A4 table, but for more complex projects, it can be elaborated on and used to generate a lengthy review article, monograph, or even book.

A final and particularly important benefit of Eight Perspectives is that the organization of perspectives can facilitate explanation of the phenomena being studied. Although the primary goal of the framework is descriptive, its organization of findings reveals connections between perspectives that otherwise might not be visible in siloed investigations. This is also a benefit of Tinbergen's Four Questions (Bateson and Laland, 2013), but in Eight Perspectives, this is enhanced and broadened, making visible connections that researchers can draw on to develop more thorough explanations of why and how such phenomena exist. For instance, despite the utility of Tinbergen's Four Questions, investigating physical attractiveness enhancement solely through the lens of the Four Questions does not allow for a comprehensive account of cultural variability (Kowal et al., 2022, 2024), nor of the emotions, cognitions, or more nuanced behavioral differences associated with this phenomenon (Arnocky et al., 2016; Davis and Arnocky, 2022; McComb and Mills, 2021).

## 7 Limitations

There are several important limitations of Eight Perspectives. First, the boundaries between perspectives are not always precise. This is a limitation of many top-down classifications, including Eight Perspectives as well as Tinbergen's Four Questions (Bateson and Laland, 2013). Researchers will need to justify their placement decisions, describe boundary cases precisely, and remain open to alternative categorizations.

A second limitation is that Eight Perspectives is descriptive and organizational by design, and true explanation will require extrapolation. Researchers who wish to use the framework to explain how and why a complex human psychobehavioral phenomenon exists or expresses the way it does will need to draw on theory and articulate the relationship between that theory and each relevant perspective. For example, a researcher applying attachment theory to romantic love would need to articulate how that theory relates to development, mechanisms, emotions, cognitions, and behavior—and in so doing, the explanatory power of the framework will be realized.

A final limitation is that this framework is not comprehensive—and indeed is not meant to be comprehensive. Just like Tinbergen's Four Questions, it attempts to describe phenomena in a more comprehensive way than a single perspective, but we expect it to fall short—as all frameworks do—because of the complexity of reality. For example, the Eight Perspectives framework describes individual-level characteristics of complex human psychobehavioral phenomena. Evidence that does not fit within the framework—such as dyadic, group, or population-level processes—may need to be considered alongside it. There will also be circumstances when subordinate psychobehavioral features will be components of the phenomenon being described, and they may warrant their own consideration separately from any particular perspective. For example, this might be the case in physical appearance enhancement with body dysmorphic disorder (Crerand et al., 2006; Higgins and Wysong, 2018) or eating disorders (Abed, 1998). The broader point is that this framework is not meant to be rigid, nor does it claim to be complete. Instead, it offers a useful starting point for researchers who wish to organize multiple strands of evidence from different fields in studying rich, complex human psychobehavioral phenomena. We hope it proves to be a useful descriptive framework for researchers from different backgrounds and scholarly traditions seeking to explain human cognition, emotion, and behavior.

## Data Availability

The original contributions presented in the study are included in the article/supplementary material, further inquiries can be directed to the corresponding author.

## References

[B1] Abed R. T. (1998). The sexual competition hypothesis for eating disorders. Br. J. Med. Psychol. 71, 525–547. doi: 10.1111/j.2044-8341.1998.tb01007.x 9875960

[B2] Al-Shawaf L. (2025). Levels of analysis and explanatory progress in psychology: integrating frameworks from biology and cognitive science for a more comprehensive science of the mind. Psychol. Rev. 132, 404–415. doi: 10.1037/rev0000459 38252109

[B3] Amir D. Pitt B. (2026). What does it mean for culture to shape cognition? Trends Cogn. Sci. doi: 10.1016/j.tics.2026.01.008 41807136

[B4] Archer J. (1999). The Nature of *Grief: The Evolution and Psychology of Reactions to Loss*. London: Routledge.

[B5] Arnocky S. Perilloux C. Cloud J. M. Bird B. M. Thomas K. (2016). Envy mediates the link between social comparison and appearance enhancement in women. Evol. Psychol. Sci. 2, 71–83. doi: 10.1007/s40806-015-0037-1

[B6] Bateson P. Laland K. N. (2013). Tinbergen's four questions: an appreciation and an update. Trends Ecol. Evol. 28, 712–718. doi: 10.1016/j.tree.2013.09.013 24144467

[B7] Bergman T. J. Beehner J. C. (2022). Leveling with Tinbergen: four levels simplified to causes and consequences. Evol. Anthropol. 31, 12–19. doi: 10.1002/evan.21931 34904769

[B8] Blake K. R. (2022). Attractiveness helps women secure mates, but also status and reproductively relevant resources. Arch. Sex. Behav. 51, 39–41. doi: 10.1007/s10508-021-01949-2 33666826

[B9] Bode A. Kushnick G. (2021). Proximate and ultimate perspectives on romantic love [Review]. Front. Psychol. 12:573123. doi: 10.3389/fpsyg.2021.573123 33912094 PMC8074860

[B10] Bode A. Kuula L. (2021). Romantic love and sleep variations: Potential proximate mechanisms and evolutionary functions. Biology 10:923. doi: 10.3390/biology10090923 34571801 PMC8468029

[B11] Brody N. (1994). Psychological science and public policy. Psychol. Inq. 5, 193–210. doi: 10.1207/s15327965pli0503_2

[B12] Bucchianeri M. M. Arikian A. J. Hannan P. J. Eisenberg M. E. Neumark-Sztainer D. (2013). Body dissatisfaction from adolescence to young adulthood: findings from a 10-year longitudinal study. Body Image 10, 1–7. doi: 10.1016/j.bodyim.2012.09.001 23084464 PMC3814026

[B13] Buss D. M. Abbott M. Angleitner A. Asherian A. Biaggio A. Blanco-Villasenor A. . (1990). International preferences in selecting mates: a study of 37 cultures. J. Cross Cult. Psychol. 21, 5–47. doi: 10.1177/0022022190211001

[B14] Chatterjee A. Thomas A. Smith S. E. Aguirre G. K. (2009). The neural response to facial attractiveness. Neuropsychology 23, 135–143. doi: 10.1037/a0014430 19254086

[B15] Crerand C. E. Franklin M. E. Sarwer D. B. (2006). Body dysmorphic disorder and cosmetic surgery. Plast. Reconstr. Surg. 118:167e. doi: 10.1097/01.prs.0000242500.28431.24 17102719

[B16] Davis A. C. Arnocky S. (2022). An evolutionary perspective on appearance enhancement behavior. Arch. Sex. Behav. 51, 3–37. doi: 10.1007/s10508-020-01745-4 33025291

[B17] d'Errico F. Vanhaeren M. Barton N. Bouzouggar A. Mienis H. Richter D. . (2009). Additional evidence on the use of personal ornaments in the Middle Paleolithic of North Africa. Proc. Nat. Acad. Sci. 106, 16051–16056. doi: 10.1073/pnas.0903532106 19717433 PMC2752514

[B18] Dixson B. J. W. (2022). Sexual selection and the evolution of human appearance enhancements. Arch. Sex. Behav. 51, 49–55. doi: 10.1007/s10508-021-01946-5 33721143

[B19] Dondzilo L. Basanovic J. Grafton B. Bell J. Turnbull G. MacLeod C. (2023). A serial mediation model of attentional engagement with thin bodies on body dissatisfaction: the role of appearance comparisons and rumination. Curr. Psychol. 42, 1896–1904. doi: 10.1007/s12144-021-01574-1

[B20] Frederick D. A. Reynolds T. A. (2022). The value of integrating evolutionary and sociocultural perspectives on body image. Arch. Sex. Behav. 51, 57–66. doi: 10.1007/s10508-021-01947-4 33751287

[B21] Geary D. C. (2000). Evolution and proximate expression of human paternal investment. Psychol. Bull. 126, 55–77. doi: 10.1037/0033-2909.126.1.55 10668350

[B22] Goodey C. F. (2006). Behavioural phenotypes in disability research: historical perspectives. J Intellect Disabil Res. 50, 397–403. doi: 10.1111/j.1365-2788.2006.00795.x 16672033

[B23] Gottesman I. I. Gould T. D. (2003). The endophenotype concept in psychiatry: etymology and strategic intentions. Am. J. Psychiatry 160, 636–645. doi: 10.1176/appi.ajp.160.4.636 12668349

[B24] Higgins S. Wysong A. (2018). Cosmetic surgery and body dysmorphic disorder—an update. Int. J. Womens Dermatol. 4, 43–48. doi: 10.1016/j.ijwd.2017.09.007 29872676 PMC5986110

[B25] Kelly R. Rose P. (2024). Sixty years of Tinbergen's four questions and their continued relevance to applied behaviour and welfare research in zoo animals: a commentary. J. Zool. Bot. Gard. 5, 338–357. doi: 10.3390/jzbg5020024

[B26] Keski-Rahkonen A. Bulik C. M. Neale B. M. Rose R. J. Rissanen A. Kaprio J. (2005). Body dissatisfaction and drive for thinness in young adult twins. Int. J. Eat. Disord. 37, 188–199. doi: 10.1002/eat.20138 15822080

[B27] Konner M. (2021). Nine levels of explanation: a proposed expansion of Tinbergen's four-level framework for understanding the causes of behavior. Hum. Nat. 32, 748–793. doi: 10.1007/s12110-021-09414-8 34739657

[B28] Kowal M. Sorokowski P. Cardona S. M. Castañeda A. Faisal C. M. N. (2024). Sex and cross-cultural comparison of self-enhancement practices: data from four distinct societies. Evol. Hum. Behav. 45:106627. doi: 10.1016/j.evolhumbehav.2024.106627

[B29] Kowal M. Sorokowski P. Pisanski K. Valentova J. V. Varella M. A. C. Frederick D. A. . (2022). Predictors of enhancing human physical attractiveness: data from 93 countries. Evol. Hum. Behav. 43, 455–474. doi: 10.1016/j.evolhumbehav.2022.08.003

[B30] Langlois J. H. Roggman L. A. Casey R. J. Ritter J. M. Rieser-Danner L. A. Jenkins V. Y. (1987). Infant preferences for attractive faces: Rudiments of a stereotype? Dev. Psychol. 23, 363–369. doi: 10.1037/0012-1649.23.3.363

[B31] Legare C. H. Nielsen M. (2020). Ritual explained: Interdisciplinary answers to Tinbergen's four questions. Philos. Trans. R. Soc. Lond. B. Biol. Sci. 375:20190419. doi: 10.1098/rstb.2019.0419 32594869 PMC7423255

[B32] Luoto S. Krams I. Rantala M. J. (2019). A life history approach to the female sexual orientation spectrum: evolution, development, causal mechanisms, and health. Arch. Sex. Behav. 48, 1273–1308. doi: 10.1007/s10508-018-1261-0 30229521

[B33] Marean C. W. Bar-Matthews M. Bernatchez J. Fisher E. Goldberg P. Herries A. I. R. . (2007). Early human use of marine resources and pigment in South Africa during the Middle Pleistocene. Nature 449, 905–908. doi: 10.1038/nature06204 17943129

[B34] Mayr E. (1961). Cause and effect in biology: kinds of causes, predictability, and teleology are viewed by a practicing biologist. Science 134, 1501–1506. doi: 10.1126/science.134.3489.1501 14471768

[B35] McComb S. E. Mills J. S. (2021). Young women's body image following upwards comparison to Instagram models: the role of physical appearance perfectionism and cognitive emotion regulation. Body Image 38, 49–62. doi: 10.1016/j.bodyim.2021.03.012 33798801

[B36] Myers T. A. Crowther J. H. (2009). Social comparison as a predictor of body dissatisfaction: A meta-analytic review. J. Abnorm. Psychol. 118, 683–698. doi: 10.1037/a0016763 19899839

[B37] Nesse R. M. (2013). Tinbergen's four questions, organized: a response to Bateson and Laland. Trends Ecol. Evol. 28, 681–682. doi: 10.1016/j.tree.2013.10.008 24216179

[B38] Nettle D. (2009). Ecological influences on human behavioural diversity: a review of recent findings. Trends Ecol. Evol. 24, 618–624. doi: 10.1016/j.tree.2009.05.013 19683831

[B39] Puts D. A. Bailey D. H. Cárdenas R. A. Burriss R. P. Welling L. L. M. Wheatley J. R. . (2013). Women's attractiveness changes with estradiol and progesterone across the ovulatory cycle. Horm. Behav. 63, 13–19. doi: 10.1016/j.yhbeh.2012.11.007 23159480

[B40] Roebroeks W. Sier M. J. Nielsen T. K. De Loecker D. Parés J. M. Arps C. E. S. . (2012). Use of red ochre by early Neandertals. Proc. Nat. Acad. Sci. 109, 1889–1894. doi: 10.1073/pnas.1112261109 22308348 PMC3277516

[B41] Simpson J. A. Belsky J. (2016). “Attachment theory within a modern evolutionary framework,” in Handbook of Attachment: Theory, Research, and Clinical Applications (3rd edn.), eds. Cassidy J. Shaver P. R. (New York, NY: Guilford Press), 91–116.

[B42] Sorokowski P. Kowal M. Sternberg R. J. Aavik T. Akello G. Alhabahba M. M. . (2023). Modernization, collectivism, and gender equality predict love experiences in 45 countries. Sci. Rep. 13:773. doi: 10.1038/s41598-022-26663-4 36641519 PMC9840424

[B43] Swami V. (2015). Cultural influences on body size ideals: unpacking the impact of Westernization and modernization. Eur. Psychol. 20, 44–51. doi: 10.1027/1016-9040/a000150

[B44] Tatangelo G. McCabe M. Mellor D. Mealey A. (2016). A systematic review of body dissatisfaction and sociocultural messages related to the body among preschool children. Body Image 18, 86–95. doi: 10.1016/j.bodyim.2016.06.003 27352102

[B45] Thompson J. K. Stice E. (2001). Thin-ideal internalization: Mounting evidence for a new risk factor for body-image disturbance and eating pathology. Curr. Dir. Psychol. Sci. 10, 181–183. doi: 10.1111/1467-8721.00144

[B46] Tinbergen N. (1963). On aims and methods of ethology. Z. Tierpsychol. 20, 410–433. doi: 10.1111/j.1439-0310.1963.tb01161.x

[B47] Tsuang M. T. Faraone S. V. Lyons M. J. (1993). Identification of the phenotype in psychiatric genetics. Eur. Arch. Psychiatry Clin. Neurosci. 243, 131–142. doi: 10.1007/BF02190719 8117756

[B48] Wang S. B. Haynos A. F. Wall M. M. Chen C. Eisenberg M. E. Neumark-Sztainer D. (2019). Fifteen-year prevalence, trajectories, and predictors of body dissatisfaction from adolescence to middle adulthood. Clin. Psychol. Sci. 7, 1403–1415. doi: 10.1177/2167702619859331 32864198 PMC7451946

[B49] Winston J. S. O'Doherty J. Kilner J. M. Perrett D. I. Dolan R. J. (2007). Brain systems for assessing facial attractiveness. Neuropsychologia 45, 195–206. doi: 10.1016/j.neuropsychologia.2006.05.009 16828125

[B50] Zietsch B. P. Sidari M. J. Murphy S. C. Sherlock J. M. Lee A. J. (2021). For the good of evolutionary psychology, let's reunite proximate and ultimate explanations. Evol. Hum. Behav. 42, 76–78. doi: 10.1016/j.evolhumbehav.2020.06.009

